# Comparative safety and effectiveness of midline catheters vs. peripherally inserted central catheters: a systematic review and meta-analysis

**DOI:** 10.3389/fcvm.2026.1890087

**Published:** 2026-08-03

**Authors:** Mohammed Aljameie, Aya Ahmed Shimal, Mohamed H. Mahmoud, Omar F. Abbas, Alaa Mohammed Oudah, Marafi Jammaa Ahmed, Khadeeja Ali Hamzah, Anas Mansour, Wael Abdullatif Abdulqader, Eathar Aljubori, Ghadeer Mohammed, Fayez Salahedin Alkhateeb, Toka Aziz El-Ramly, Alhareth Al-Sagban, Howard Graham, Maria Carolina Musri

**Affiliations:** 1 Hammurabi College of Medicine, University of Babylon, Babil, Iraq; 2 College of Medicine, University of Baghdad, Baghdad, Iraq; 3 Faculty of Medicine, Alexandria University, Alexandria, Egypt; 4 Faculty of Medicine, Al-Azhar University, Cairo, Egypt; 5 Al-Kindy Medical College, University of Baghdad, Baghdad, Iraq; 6 Faculty of Medicine, Bahri University, Khartoum, Sudan; 7 Kufa University, College of Medicine, Najaf, Iraq; 8 Faculty of medicine, University of Jordan, Amman, Jordan; 9 College of Medicine, University of Mosul, Mosul, Iraq; 10 Johns Hopkins University-School of Medicine, Baltimore, MD, United States

**Keywords:** complications, meta-analysis, midline catheter (MC), PICC catheter, safety

## Abstract

**Background & objective:**

Peripherally inserted central catheters (PICCs) and midline catheters (MCs) are widely used for intravenous therapy, but their relative safety and effectiveness remain uncertain. This systematic review and meta-analysis aimed to compare complication rates between PICCs and MCs.

**Methods:**

We systematically searched PubMed, Scopus, Embase, and Web of Science for randomized controlled trials (RCTs) and observational studies evaluating PICCs vs. MCs. Risk of bias was assessed using the Cochrane RoB 2 tool for RCTs and the Newcastle-Ottawa Scale for observational studies. Meta-analyses were performed to calculate pooled risk ratios (RRs) and standardized mean differences (SMDs) with 95% confidence intervals (CIs).

**Results:**

Fifteen studies (3 RCTs and 12 cohort studies) including over 27,000 patients were analyzed. PICCs were associated with a significantly higher risk of CRBSIs compared with MCs (RR = 4.18, 95% CI: 2.87–6.08; *p* < 0.01). No significant differences were observed between the two devices in rates of deep vein thrombosis (DVT), pulmonary embolism (PE), device failure, occlusion, dislodgement, catheter migration, phlebitis, or mortality. PICCs demonstrated longer dwell times (SMD = 0.68; 95% CI: 0.18–1.07; *p* < 0.01), consistent with their use in prolonged therapies.

**Conclusion:**

MCs are associated with a lower risk of bloodstream infections compared with PICCs. PICCs remain appropriate for long-term therapy, while MCs may represent a safer alternative for shorter courses. Device selection should be individualized based on patient risk factors, treatment duration, and clinical context.

## 1 Introduction

Intravenous (IV) access is one of the most common invasive procedures in hospitalized patients and remains a cornerstone of modern medical care (1). Reliable venous access allows clinicians to deliver medications, fluids, blood products, and parenteral nutrition across a wide range of clinical situations (2). For patients who need longer courses of IV therapy, two devices are commonly used: the peripherally inserted central catheter (PICC) and the midline catheter (MC) (3).

A PICC is a long catheter placed into a peripheral vein of the upper arm, with its tip advanced into a central vein, usually at the atrio-caval junction near the right atrium (3). Because of its central placement, a PICC can be used for irritant or vesicant drugs, chemotherapy, and parenteral nutrition, making it highly versatile for prolonged treatment (4, 5). In contrast, an MC is shorter. It is also inserted in the upper arm, but its tip ends in a large peripheral vein at or just before the axilla, outside the major thoracic vessels (6). MCs are generally recommended for therapies lasting one to four weeks, particularly for patients with difficult venous access, though their use is usually limited to peripherally compatible infusions (2).

Both devices have become more common because they are relatively easy to insert and provide reliable access. However, neither is without risk (7). Like other central lines, PICCs can cause serious complications, including catheter-related bloodstream infections (CRBSIs), thrombosis (CRT), and occlusion (6). These problems may result in device failure, treatment interruptions, higher costs, and longer hospital stays (8). To reduce such complications, especially CRBSI, many clinicians have turned to MCs as a potentially safer alternative (9). This trend is supported by guidelines such as the Michigan Appropriateness Guide for Intravenous Catheters (MAGIC), which often recommend MCs over PICCs for certain short-term needs (10).

Despite these recommendations, there is still no clear consensus on which device is safer or more effective. Some studies and meta-analyses suggest that MCs carry a lower risk of CRBSI and occlusion, while others report equal or even higher complication rates, including thrombosis and premature device failure. Recently, Tian et al. conducted an exposure-adjusted meta-analysis evaluating MCs vs. PICCs, focusing heavily on incidence rate ratios (IRR) (11). While their findings provide valuable insights into time-adjusted complication rates across 11 studies, the present study extends this evidence base by incorporating a significantly larger cohort of over 27,000 patients across 15 studies. Furthermore, our analysis evaluates distinct and critical secondary outcomes, such as local exit-site infections, device failure, and catheter occlusion, while performing robust subgroup analyses stratifying RCTs vs. observational data to isolate high-certainty evidence.

The purpose of this meta-analysis is to systematically evaluate the evidence comparing MCs and PICCs. By focusing on key outcomes such as CRBSI, thrombosis, occlusion, and overall device failure, this study aims to clarify the relative risks and benefits of each device and to guide clinicians toward safer and more effective vascular access decisions in everyday practice.

## 2 Methodology

### 2.1 Protocol registration

The present systematic review and meta-analysis were conducted in accordance with the Preferred Reporting Items for Systematic Reviews and Meta-Analyses (PRISMA) guidelines (12). The PRISMA checklist was followed to ensure methodological rigor.

### 2.2 Data sources & search strategy

A literature search was conducted in PubMed, Scopus, Embase, and Web of Science. The subject terms were as follows: (“Catheterization, Central Venous” [MeSH] OR “Catheters, Indwelling” [MeSH] OR “Infusions, Intravenous” [MeSH] OR “peripherally inserted central catheter” OR “PICC” OR “central venous catheter”) AND (“midline catheter” OR “midline venous catheter” OR “Catheters”). A broad search filter was applied to identify all studies; synonyms to the terms used were found on Medical Subject Headings (MeSH), and the terms were connected by “OR” and “AND” operators from the Cochrane Handbook for Systematic Reviews.

### 2.3 Eligibility criteria and selection process

Studies were considered eligible according to the PICOS framework. We included randomized controlled trials and observational cohort studies that directly compared peripherally inserted central catheters (PICCs) with midline catheters (MCs) among patients requiring intravenous vascular access for medical treatment in either inpatient or outpatient clinical settings. The study population included hospitalized adults, intensive care unit (ICU) patients, oncology patients, and other clinical populations receiving intravenous therapy; studies involving both adult and pediatric populations were eligible if outcomes for PICCs and MCs were reported separately. Eligible studies were required to report at least one catheter-related outcome, including catheter-related bloodstream infection (CRBSI), deep vein thrombosis (DVT), pulmonary embolism (PE), catheter occlusion, device failure, catheter dislodgement, catheter migration, phlebitis, exit-site infection, or other catheter-related complications. Studies were excluded if they did not include both PICC and MC comparison groups, if outcome data for the two devices were not reported separately, or if they were non-original articles such as case reports, case series with fewer than 10 patients, reviews, conference abstracts, editorials, letters, or animal studies.

Two reviewers independently screened titles and abstracts using Rayyan software, followed by full-text assessment to determine eligibility. Any disagreements were resolved through discussion or consultation with a third reviewer.

### 2.4 Data extraction

Two authors extracted the data independently. The data collected included the first author's name, year of publication, study population, sample size, age, gender distribution, follow-up period, BMI, and initial morbidity. Primary outcomes included CRBSI. Secondary outcomes included catheter occlusion, DVT, dwelling time, dislodgment, device failure, catheter migration, local exit site infection, mortality, phlebitis, and pulmonary embolism.

### 2.5 Definitions of complications

To ensure consistency across the included studies, standard clinical definitions were applied where reported. Catheter-Related Bloodstream Infection (CRBSI) was defined as bacteremia or fungemia in a patient with an intravascular catheter with at least one positive blood culture. Deep Vein Thrombosis (DVT) was defined as symptomatic or asymptomatic thrombosis confirmed by ultrasound. Catheter occlusion was defined as the inability to infuse fluids or draw blood. Device failure encompassed any complication leading to premature catheter removal. Phlebitis was defined as vein inflammation characterized by pain, erythema, or swelling.

### 2.6 Risk of bias and certainty of evidence

Two independent reviewers conducted a risk of bias assessment for the studies included in the analysis. For randomized controlled trials (RCTs), the Cochrane Risk of Bias 2 (ROB2) tool was utilized to assess domains including the randomization process, deviations from intended interventions, missing outcome data, measurement of outcomes, and selection of reported results (13). For observational studies, the Risk Of Bias In Newcastle-Ottawa Scale (NOS) assessment tool was applied (14). This tool assesses observational studies in three domains: selection, comparability, and outcomes. Each study earned an overall score ranging from 0 to 9, with scores of 3 or less indicating poor quality, 4–6 suggesting fair quality, and 7 or more indicating good quality.

### 2.7 Statistical analysis

For dichotomous outcomes, the number of events and the total number of patients in each group were combined to calculate the risk ratio (RR) with a 95% confidence interval (CI) using a random-effects model. Continuous outcomes such as dwelling time were analyzed using Standardized Mean Differences (SMD). Meta-analysis was performed using R software version 4.4.3. Statistical heterogeneity across studies was assessed using Cochran's *Q* test and quantified by the *I*^2^ statistic. An *I*^2^ value of more than or equal to 50 indicates heterogeneity. A *p*-value of <0.10 in the *Q* test was regarded as indicative of significant heterogeneity.

To assess the robustness of our findings, a leave-one-out sensitivity analysis was performed by iteratively removing one study at a time and recalculating the pooled effect estimate to identify whether any single study disproportionately influenced the overall results. Where ≥10 studies were available, we assessed small-study effects and publication bias using funnel plots, as recommended by the Cochrane Handbook for Systematic Reviews of Interventions, which notes that analyses with fewer than 10 studies lack sufficient statistical power to distinguish true asymmetry from chance (15).

## 3 Results

### 3.1 Search results and study selection

**Figure 1:**
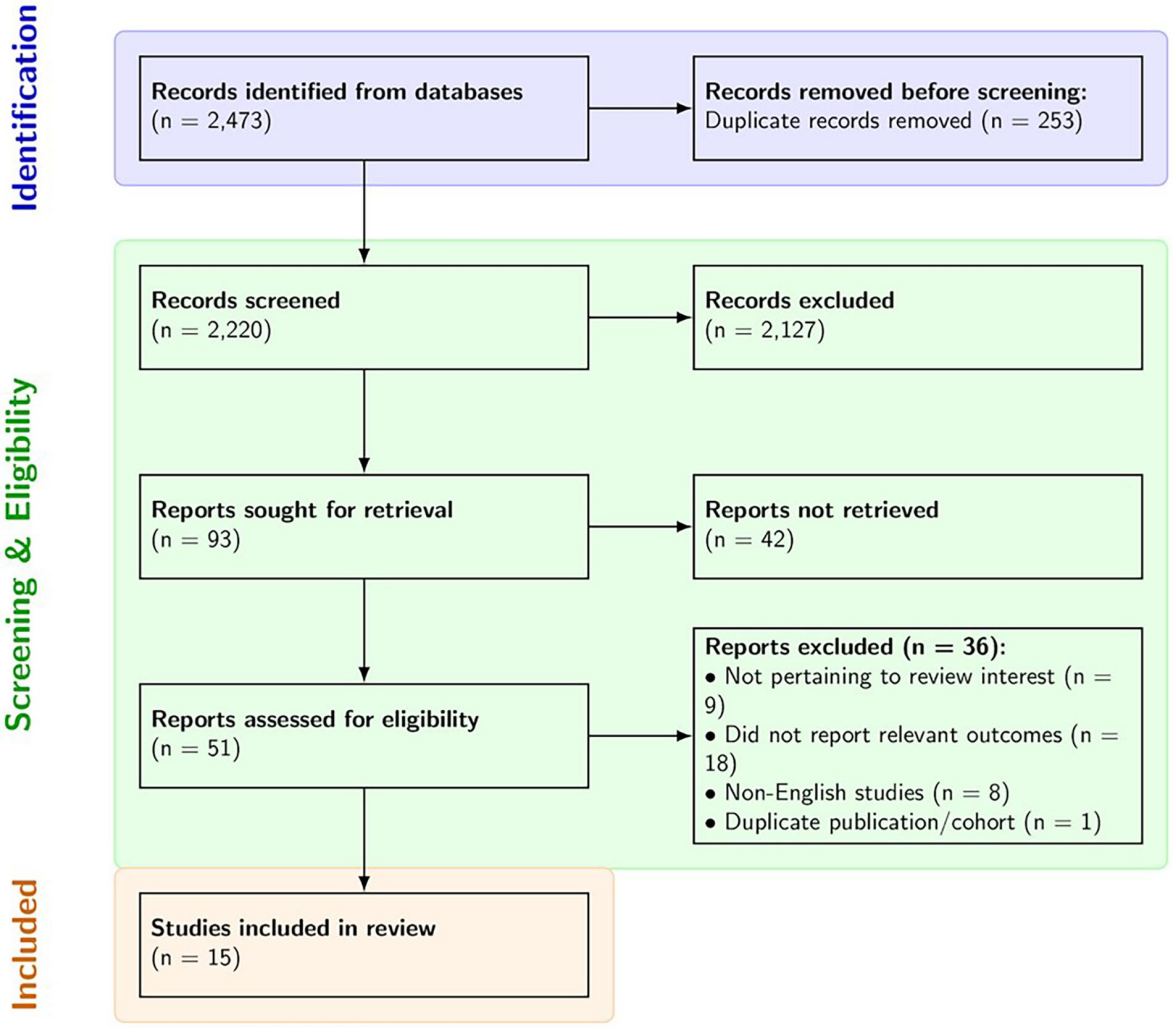
PRISMA flow chart of the screening process.

The search yielded 2,473 records. After removing 253 duplicates, 2,220 records were screened, of which 2,127 were excluded based on title and abstract review. A total of 93 reports were sought for full-text retrieval, 42 were not retrieved, and 51 were assessed for eligibility. 36 studies were excluded, and 15 studies were included in the systematic review and meta-analysis (Figure 1).

### 3.2 Characteristics of included studies

A total of 15 studies (3 RCTs and 12 cohort studies) (2–7, 9, 10, 16–23) encompassing over 27,000 participants were included in the analysis. Sample sizes ranged from 106 to 10,863 patients. The mean age of participants spanned from the early 60s to the late 70s. Most studies originated from the United States (*n* = 7), followed by Europe (*n* = 5) as well as Australia (*n* = 1), Canada (*n* = 1), and India (*n* = 1). The baseline and summary characteristics are illustrated in Tables 1, 2.

**Table 1:** Summary characteristics of the included studies.

Study ID	Country	Study design	Study period	Main conclusion	Inclusion criteria
Faccini, 2024	Italy	Prospective observational	2019–2022	PICC preferred over MC for majority of chemotherapy	Adult cancer patients ≥16 years
Grigg, 2024	United States	Retrospective chart review	2021–2022	No difference between CRBSI, thrombosis rates	Hospitalized patients receiving vesicant medications
Kleidon, 2021	Australia	Randomized controlled trial	2019–2020	Feasibility of efficacy trial to compare MC to PICC	Children 1 to <18 years
Bahl, 2019	United States	Retrospective chart review	2016–2017	Symptomatic CR thrombosis more frequent in MCs	Adults >18 years with MC or PICC
Frondizi, 2023	Italy	Prospective observational	2020–2021	No significant differences in infection rates	Non-ICU COVID-19 patients
Rosich-Soteras, 2023	Spain	Longitudinal descriptive	2019–2020	VAT enabled safe/effective insertion, low complications	Patients ≥18 years
Bentridi, 2025	Canada	Randomized Clinical Trial	2018–2022	MVCs associated with higher adverse events/dysfunction	Adult patients referred for PICC insertion
Paje, 2025	United States	Retrospective cohort	2017–2023	MCs are safe alternatives to PICCs for OPAT (≤14 days)	Patients receiving antimicrobial therapy via MC/PICC
Bing, 2022	United States	Retrospective review	2014–2016	MC may not decrease overall CRBSI/thrombosis incidences	Patients receiving MC or PICC
Swaminathan, 2022	United States	Cohort study	2017–2020	PICCs associated with higher risk of major complications	Hospitalized adults receiving PICCs or midlines (≤30 days)
Thomsen, 2024	Denmark	Randomized clinical trial	2018–2022	No significant difference in CRBSI; MCs as alternative	Adults requiring IV therapy for 5–28 days
Raghunathan, 2023	India	Retrospective observational	2019–2021	MCs functionally similar to PICCs, lesser complications	Pediatric ICU patients
Xu, 2016	United States	Retrospective study	2015–2015	MCs are an acceptable alternative to reduce CLABSIs	Hospitalized patients receiving Bard PICCs/MCs
Gravdahl, 2023	Norway	Retrospective study	2020–2022	PICC and MC are safe/efficient for palliative cancer care	Adult cancer patients in Palliative Medicine
Lescinskas, 2020	United States	Prospective observational	2015–2017	MCs effective for short-term access; PICC for chemo	Patients ≥18 years with new MC/PICC within 3 days

**Table 2:** Baseline characteristics of the participants.

Study ID	Total participants (n)	Male, *n* (%)	Age, years (Mean ± SD)	BMI (Mean ± SD)	Race/ethnicity	Key comorbidities
Faccini, 2024	555	246 (44.3%)	60.05 ± 12.22	—	—	—
Grigg, 2024	445	—	—	—	—	—
Kleidon, 2021	106	46 (43.4%)	—	—	—	Cystic fibrosis, bronchiectasis, prior VTE/occlusion
Bahl, 2019	2,577	1,177 (45.7%)	63.7 ± 17.28	—	—	History of DVT or PE
Frondizi, 2023	227	95 (41.9%)	78.08 ± 13.49	—	—	Hypertension, CVD, diabetes, lung disease, obesity
Rosich-Soteras, 2023	1,056	650 (61.6%)	MC: 66.6 ± 15.3 PICC: 61.8 ± 14.5	—	—	—
Bentridi, 2025	272	—	—	—	Mixed (White, Black, Asian, Native American, Other)	—
Paje, 2025	2,824	1,487 (52.7%)	—	—	Black, White, Other/Unknown	CKD, hemodialysis, malignancy, liver disease, prior CLABSI/VTE
Bing, 2022	3,560	1,708 (48.0%)	—	—	Hispanic, Non-Hispanic	—
Thomsen, 2024	304	174 (57.2%)	MC: 64.4 ± 14.1 PICC: 64.8 ± 12.9	MC: 28.1 ± 5.9 PICC: 27.7 ± 7.0	—	CVD, pulmonary disease, diabetes, renal/liver disease, malignancy
Raghunathan, 2023	265	200 (75.5%)	—	—	—	—
Swaminathan, 2022	10,863	5,122 (47.1%)	—	—	Asian, Black, White	Renal failure, CVD, pulmonary disease, diabetes, malignancy, sepsis
Xu, 2016	367	192 (52.3%)	—	—	—	—
Gravdahl, 2023	374	188 (50.3%)	—	—	—	—
Lescinskas, 2020	113	65 (57.5%)	MC: 49.1 ± 12.9 PICC: 45.5 ± 13.9	—	White, Black, Hispanic, Other	—

MC, Midline Catheter; PICC, Peripherally Inserted Central Catheter; SD, standard deviation; BMI, body mass index; CVD, cardiovascular disease; VTE, venous thromboembolism; PE, pulmonary embolism; DVT, deep vein thrombosis; CKD, chronic kidney disease; CLABSI, Central Line-Associated Bloodstream Infection.

“—” indicates data not reported.

### 3.3 Risk of bias and certainty of evidence

**Figure 2:**
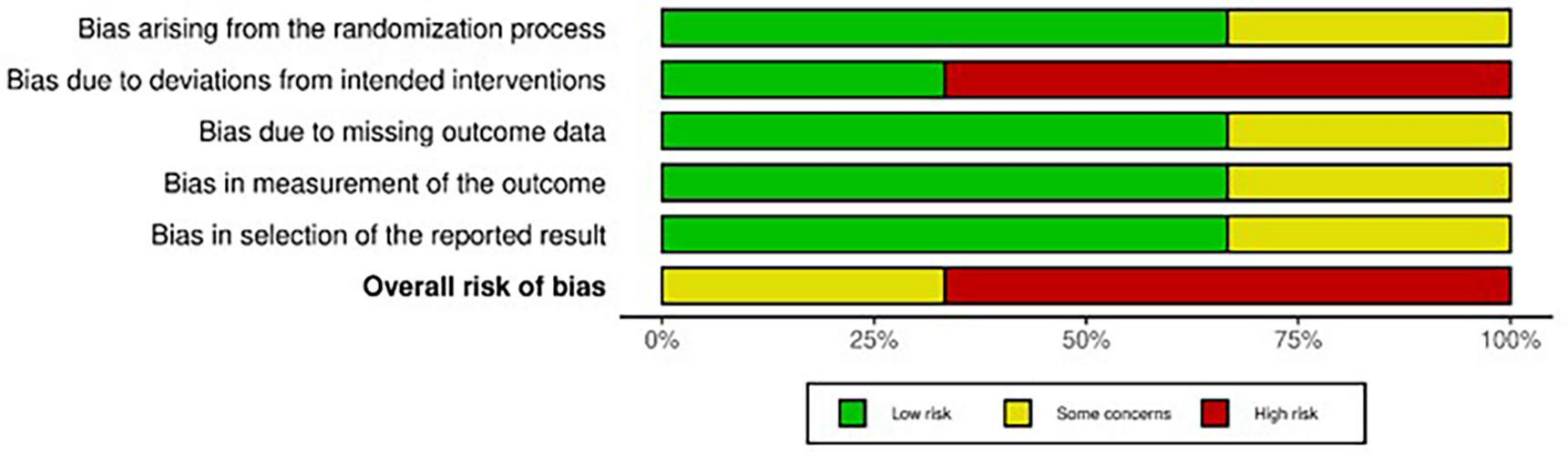
Risk of bias assessment of RCTs.

**Figure 3:**
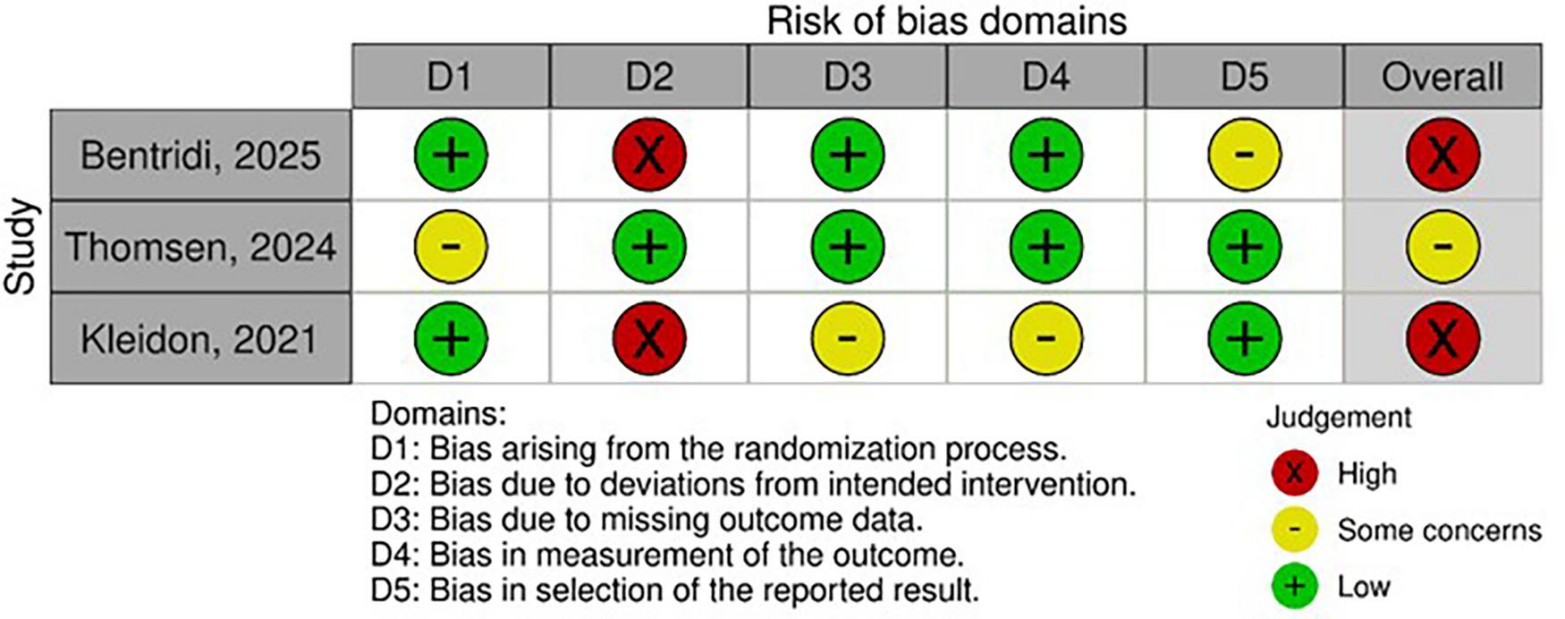
Summary of the risk of bias of RCTs.

Among the RCTs, one was judged to have a high risk of bias, while the remaining two were rated as having some concerns. Most cohort studies demonstrated good quality, with one study (Frondizi 2023) indicating fair quality (NOS score 6/9) (Table 3; Figures 2 and 3).

**Table 3:** Newcastle-Ottawa scale (NOS) quality assessment.

Study	Selection	Comparability	Exposure/outcome	Total score
Swaminathan, 2022	4 stars	2 stars	3 stars	9/9
Paje, 2025	4 stars	2 stars	2 stars	8/9
Faccini, 2024	4 stars	1 stars	3 stars	8/9
Frondizi, 2023	4 stars	0 stars	2 stars	6/9
Lescinskas, 2020	4 stars	1 stars	3 stars	8/9
Raghunathan, 2023	4 stars	1 stars	3 stars	8/9
Xu, 2016	4 stars	1 stars	3 stars	8/9
Gravdahl, 2023	4 stars	1 stars	3 stars	8/9
Rosich-Soteras, 2023	4 stars	1 stars	3 stars	8/9
Bing, 2022	4 stars	1 stars	3 stars	8/9
Grigg, 2024	4 stars	2 stars	3 stars	9/9
Bahl, 2019	4 stars	2 stars	3 stars	9/9

### 3.4 GRADE certainty of evidence assessment

The certainty of evidence regarding the comparison between PICCs and MCs showed that CRBSI, DVT, and catheter occlusion were supported by very low-certainty evidence. All outcomes started as low certainty as the majority of studies were observational studies. Crucially, no serious publication bias was detected in the funnel plot assessments for CRBSI, DVT, and occlusion (Table 4).

**Table 4:** GRADE certainty of evidence.

Outcome	No. Studies	Risk of Bias	Inconsistency	Imprecision	Publication Bias	Certainty
CRBSI	11	Not serious	Not serious	Not serious	Not serious	Very Low
Deep Vein Thrombosis (DVT)	12	Not serious	Serious	Serious	Not serious	Very Low
Catheter Occlusion	11	Not serious	Serious	Not serious	Not serious	Very Low

### 3.5 Primary outcome: CRBSIs

Our analysis encompassed 8,914 patients in the PICC group and 8,673 in the MC group. Meta-analysis showed a significantly higher risk of Catheter-Related Bloodstream Infections with PICC (RR = 4.18, 95% CI: 2.87–6.08; *P* < 0.01), with no observed heterogeneity (*I*^2^ = 0%, *P* = 0.84). A leave-one-out sensitivity test was performed, confirming that no individual study had a disproportionate impact on the pooled estimate. Subgroup analysis based on study design revealed that among randomized controlled trials (RCTs), the difference was not statistically significant, and the results were homogeneous. In contrast, cohort studies demonstrated a significantly higher risk in the PICC group (RR = 4.30, 95% CI: 2.93–6.31; *P* < 0.01), with similarly homogenous results (*I*^2^ = 0%, *P* = 0.72) (Supplementary Figures S1, S2).

### 3.6 Secondary outcomes

The meta-analysis revealed a significant difference in dwelling time between the two groups, favouring MCs (SMD = 0.68; 95% CI: 0.18–1.07; *p* < 0.01), accompanied by substantial heterogeneity (*I*^2^ = 97%; *p* < 0.01). Leave-one-out analysis showed no single study effect on the pooled estimate or heterogeneity. Subgroup analysis among RCTs revealed a statistically significant lower dwelling time among the MC group (SMD = 0.24; 95% CI: 0.00–0.48; *p* = 0.05), with decreasing heterogeneity (*I*^2^ = 53%; *p* = 0.12). Cohort studies also showed a significant difference, but with persistent high heterogeneity (Supplementary Figures S3–S5).

For local exit-site infection, the PICC group demonstrated a significantly lower risk (RR = 0.32; 95% CI: 0.17–0.61; *p* = 0.01), with no observed heterogeneity (*I*^2^ = 0%; *P* = 0.72). Sensitivity analysis excluding Paje 2025 showed no significant difference (RR = 0.69; 95% CI: 0.08–6.11), while homogeneity was maintained (Supplementary Figures S6, S7).

Regarding phlebitis, a significantly higher risk was noted in the PICC group (RR = 2.97; 95% CI: 1.06–8.29; *p* = 0.04), with no heterogeneity (*I*^2^ = 0%; *p* = 0.50). However, excluding Raghunathan 2023 in the sensitivity analysis made the result non-significant (RR = 1.24; 95% CI: 0.31–4.97) (Supplementary Figures S8, S9).

Pulmonary embolism showed no significant difference between groups (RR = 1.36; 95% CI: 0.46–4.05; *p* = 0.58), with moderate heterogeneity (*I*^2^ = 64%; *P* = 0.04). Excluding Bahl 2019 reduced heterogeneity to 33% and adjusted the pooled RR to 2.58 (95% CI: 0.53–12.62), though the result remained non-significant (Supplementary Figures S10, S11).

For deep vein thrombosis (DVT), no significant difference was observed (RR = 1.00; 95% CI: 0.69–1.44; *p* = 0.99), with high heterogeneity (*I*^2^ = 71%; *p* < 0.01). Sensitivity analysis excluding Bahl 2019 eliminated heterogeneity (*I*^2^ = 0%) and yielded a pooled RR of 1.16 (95% CI: 0.95–1.41), still non-significant (Supplementary Figures S12, S13).

Mortality rate showed no significant difference (RR = 0.41; 95% CI: 0.06–2.68; *p* = 0.25), with considerable heterogeneity (*I*^2^ = 94%; *p* < 0.01) (Supplementary Figures S14). Dislodgement also showed no significant difference (RR = 1.26; 95% CI: 0.94–1.70; *p* = 0.13), with no heterogeneity (*I*^2^ = 0%; *p* = 0.44). Also, leave-one-out revealed that no single study influenced the pooled estimate (Supplementary Figures S15, S16).

For device failure, no significant difference was found (RR = 0.86; 95% CI: 0.54–1.39; *p* = 0.55), and high heterogeneity was present (*I*^2^ = 82%; *p* < 0.01). Sensitivity analysis showed no impact on heterogeneity or significance. Subgroup analysis revealed that among randomized controlled trials (RCTs), the PICC group had a significantly lower risk (RR = 0.41; 95% CI: 0.23–0.74; *p* < 0.01), with no heterogeneity (*I*^2^ = 0%; *p* = 0.45), whereas cohort studies showed persistent heterogeneity and no significant difference (Supplementary Figures S17–S19).

Catheter occlusion similarly showed no overall difference (RR = 1.07; 95% CI: 0.53–2.16; *p* = 0.86), with high heterogeneity (*I*^2^ = 90%; *p* < 0.01). Sensitivity analysis had no effect, but subgroup analysis among RCTs revealed a significantly lower risk in the PICC group (RR = 0.28; 95% CI: 0.15–0.52; *p* < 0.01) with low heterogeneity (*I*^2^ = 9%; *P* = 0.33). Cohort studies again showed no significant difference and high heterogeneity (Supplementary Figures S20–S22).

For catheter migration, no significant difference was observed (RR = 0.66; 95% CI: 0.53–2.16; *P* = 0.86), with high heterogeneity (*I*^2^ = 72%; *p* < 0.01). However, excluding Paje 2025 in the sensitivity analysis revealed a significantly lower risk in the PICC group (RR = 0.26; 95% CI: 0.07–0.96), with homogeneity restored (*I*^2^ = 0%) (Supplementary Figures S23, S24).

### 3.7 Publication bias

Publication bias was assessed for outcomes reported by 10 or more studies. For catheter-related bloodstream infections, the funnel plot appeared symmetrical, and Egger's test supported this with non-significant findings (*P* = 0.2565). The same was true for DVT, with a symmetrical funnel plot and non-significant Egger's test results (*P* = 0.2565). Similarly, for catheter occlusion, the funnel plot was symmetrical, and Egger's test also supported this with non-significant findings (*P* = 0.1356) (Supplementary Figures S25–S27).

## 4 Discussion

This systematic review and meta-analysis compared midline catheters (MCs) with peripherally inserted central catheters (PICCs) across more than 27,000 patients from diverse populations and clinical settings. Our findings provide clinically meaningful insights into the relative safety and effectiveness of these devices in contemporary practice.

The most noteworthy finding in our meta-analysis was the considerably higher risk of catheter-related bloodstream infections associated with PICCs. Across 8,914 PICC patients and 8,673 midline patients, CRBSI occurred in 146 (1.6%) of PICC patients vs. 52 (0.6%) of midline patients, corresponding to a pooled RR 4.18 (95% CI: 2.87–6.08; *p* < 0.01) with no observed heterogeneity (*I*^2^ = 0%). Subgroup analysis confirmed that this effect was primarily driven by observational studies, while the limited RCT evidence did not reach statistical significance, highlighting the need for more high-quality trials.

These findings are particularly relevant as most included cohorts were older adults (mean age 60–70 years) and frequently cancer patients, populations already predisposed to infection. Moreover, many PICC groups used multi-lumen polyurethane catheters, which are known to elevate CRBSI risk compared with the single-lumen MCs used in several studies. This risk factor was also reflected in previous literature (24, 25).

A previous meta-analysis (26) reported that the midlines might experience fewer CRBSIs than PICCs, reinforcing the notion that remaining outside the central circulation could reduce the opportunity for intraluminal colonization and subsequent bloodstream infection (12). A separate study demonstrated that PICCs were associated with significantly lower CLABSI rates compared with centrally inserted CVCs, despite their longer dwell times (27). In contrast, another systematic review found that the prevalence of CRBSI did not differ significantly between midline catheters (MCs) and PICCs, suggesting that both devices may carry comparable risks when used in similar populations and durations of therapy (28). These discrepant findings likely reflect important contextual differences. In patients at high risk of infection, such as those with cancer, advanced age, or extended hospitalization, MCs may be a safer option than PICCs, lowering CRBSI incidence. PICCs continue to be useful for individuals who require long-term vascular access. Thus, device selection should be individualized, balancing patient profile, treatment duration, and institutional expertise.

Thrombotic outcomes such as DVT and pulmonary embolism showed no significant difference, countering earlier assumptions that PICCs inherently carry a higher thrombosis risk. Though many included cohorts had high baseline risk, up to 40%–50% male representation, and prior VTE history in some populations, likely influencing thrombotic outcomes irrespective of device type. These observations are consistent with recent evidence. A 2024 meta-analysis of randomized trials reported that PICCs and midlines had comparable thrombosis rates, with PICCs showing fewer total complications and longer dwell times in some pooled analyses (1).

Other outcomes provided important context for selecting the device. PICCs were associated with significantly longer dwell times. Consistent with the intended use, PICCs in the included studies were commonly chosen for chemotherapy and prolonged IV therapy, while MCs were more often used for antibiotics or difficult access, explaining the shorter durations. Meta-analyses confirm this duration difference and note that MCs are often removed prematurely, reinforcing how device selection aligns with treatment goals (1). This supports their established role in long-term therapy, but may also contribute to their higher infection risk (29, 30).

Regarding local catheter-related complications, PICCs were associated with a lower risk of exit-site infections (RR = 0.32; 95% CI: 0.17–0.61; *p* = 0.01), indicating a potential protective effect. However, sensitivity analysis excluding Paje 2025 rendered this difference non-significant, suggesting that individual studies influence pooled estimates. Conversely, the risk of phlebitis was higher in the PICC group (RR = 2.97; 95% CI: 1.06–8.29; *P* = 0.04), although this became non-significant when Raghunathan 2023 was excluded. These findings highlight a nuanced risk-benefit profile for PICCs, which may reduce exit-site infections while increasing phlebitis risk in certain contexts.

Secondary outcomes, including device failure, occlusion, dislodgement, migration, and mortality, did not differ significantly between groups. The pooled risk ratios were: device failure 0.86 (95% CI: 0.54–1.39), occlusion 1.07 (95% CI: 0.53–2.16), dislodgement 1.26 (95% CI: 0.94–1.70), migration 0.66 (95% CI: 0.53–2.16), and mortality 0.41 (95% CI: 0.06–2.68). Although these outcomes showed no clear differences, the analyses were limited by high heterogeneity and, in some cases, sensitivity to single studies, underscoring the uncertainty around these estimates.

A review assessed 17 studies on catheter utilization in palliative care cancer patients. It reported minimal complication rates, and the incidence of thrombosis, infections, and occlusion ranged from 0 to 2.46 per 1,000 catheter days. The study highlighted the safety and value of both PICCs and MCs in palliative care settings (31). This further supports our finding that in cancer populations, both devices can be used safely with minimal complication rates, provided careful monitoring and appropriate selection.

Overall, our review emphasizes the complexity of device selection and requirement for aligning evidence with patient needs, while making a contribution to the growing evidence base underpinning vascular access selection in many different care settings.

### 4.1 Limitations and future perspectives

This review has several limitations. Most of the included studies were observational, with few randomized controlled trials, and consequently may have introduced bias and limited causal inference. Considerable heterogeneity across patient populations, catheter types, and clinical settings may also have influenced the pooled estimates. In addition, some outcomes were also guided by low numbers of studies, reducing statistical power and precision. Despite the use of an extensive search strategy, publication bias is not ruled out entirely.

Furthermore, while our review encompasses a large patient cohort, it is important to explicitly emphasize the statistical fragility of several secondary outcomes. In our leave-one-out sensitivity analyses, outcomes such as local exit-site infections, phlebitis, and catheter migration lost statistical significance upon the exclusion of single studies. Consequently, these specific findings lack robustness and should be interpreted with high clinical caution. They currently cannot form the basis of strong clinical recommendations until corroborated by further high-quality randomized trials.

Prospective high-quality multicenter randomized studies that compare MCs and PICCs within more homogeneous populations must be the direction taken by future research. Studies exploring cost-effectiveness, patient-reported outcomes, and the role of vascular access teams are also needed to guide practice and policy.

## 5 Conclusion

This systematic review and meta-analysis made the conclusion that MCs is associated with a lower risk of bloodstream infections compared with PICCs, but overall complication and thrombotic rates are similar. These findings emphasize the importance of choosing individual vascular access depending on patient risk factors and treatment requirements.

## Data Availability

The original contributions presented in the study are included in the article/Supplementary Material, further inquiries can be directed to the corresponding author.
