# Supplementary material for: Comparative safety and effectiveness of midline catheters vs. peripherally inserted central catheters: a systematic review and meta-analysis
Source: Front Cardiovasc Med. 2026 Aug 3;13:1890087. doi: 10.3389/fcvm.2026.1890087 (PMC13477894; doi:10.3389/fcvm.2026.1890087)
Supplement: Supplementary file 1 [file Datasheet1.docx]

**Supplementary figures:**

**Figure S1:**CRBSI Forest plot across included studies

displaying individual and pooled effect estimates with 95% confidence intervals.

**Figure S2**. CRBSI subgroup analysis rates to evaluate differences across study-level variables.

**Figure S3**. dwelling time forest plot comparing catheter dwelling time between groups. Mean differences with 95% confidence intervals are presented.

**Figure S4**. dwelling time subgroup based on study characteristics.

**Figure S5:**. dwelling time leave-one-out evaluating the influence of each study on pooled dwelling time results.

**Figure S6**. local-exist-site-reaction forest plot showing the incidence of local exit-site reactions across studies with pooled effect estimate and 95% confidence intervals.

**Figure S7**. Catheter occlusion leave one out sensitivity analysis for catheter occlusion, assessing the stability of pooled results.

**Figure S8.**. Phlebitis forest plot presenting the incidence of phlebitis across studies, including pooled estimate and 95% confidence intervals.

**Figure S9.**. Phlebitis leave one out Sensitivity analysis assessing robustness of device failure results.

**Figure S10.**. Pulmonary embolism forest plot of pulmonary embolism incidence across studies with pooled estimate.

**Figure S11.**. Pulmonary embolism leave one out sensitivity analysis for pulmonary embolism outcomes.

**Figure S12**. .DVT forest plot Forest plot showing deep vein thrombosis (DVT) incidence across studies with pooled effect estimate.

**Figure S13**. DVT leave one out Sensitivity analysis

**Figure S14**. .mortality rate forest plot presenting mortality rates across studies with pooled estimates and 95% confidence intervals.

**Figure S15**. Dislodgment forest plot rates across studies with pooled estimate.

**Figure S16**. Dislodgment Leave-one-out sensitivity analysis for catheter dislodgment.

**Figure S17** . Device failure forest plot showing device failure rates across studies with pooled estimate.

**Figure S18**. .Device failure leave one out Sensitivity analysis assessing robustness of device failure results.

**Figure S19**. Device failure Subgroup analysis exploring heterogeneity in device failure rates.

**Figure S20.**. Catheter occlusion forest plot showing catheter occlusion rates across studies with pooled estimate.

**Figure S21.**. Catheter occlusion subgroup analysis examining differences in catheter occlusion rates.

**Figure S22**. Catheter occlusion Leave-one-out sensitivity analysis for catheter occlusion.

**Figure S23**. Catheter migration forest plot presenting catheter migration incidence across studies with pooled estimate.

**Figure S24**. Catheter migration leave one out Sensitivity analysis

**Figure S25**. Funnel plot assessing publication bias for CRBSI outcomes. Symmetry suggests low risk of bias, while asymmetry may indicate potential bias.

**Figure S26**. Funnel plot evaluating publication bias for DVT outcomes.

**Figure S27**. Funnel plot assessing publication bias for catheter occlusion outcomes.
